# Flame Retardance and Antistatic Polybutylene Succinate/Polybutylene Adipate-Co-Terephthalate/Magnesium Composite

**DOI:** 10.3390/polym17121675

**Published:** 2025-06-17

**Authors:** Pornchai Rachtanapun, Jonghwan Suhr, Eunyoung Oh, Nanthicha Thajai, Thidarat Kanthiya, Krittameth Kiattipornpithak, Kannikar Kaewapai, Siriphan Photphroet, Patnarin Worajittiphon, Nuttapol Tanadchangsaeng, Pitiwat Wattanachai, Kittisak Jantanasakulwong, Choncharoen Sawangrat

**Affiliations:** 1 Faculty of Agro-Industry, Chiang Mai University, Chiang Mai 50100, Thailand; pornchai.r@cmu.ac.th (P.R.); siriphan_photp@cmu.ac.th (S.P.); 2 School of Mechanical Engineering, Sungkyunkwan University, 2066 Seobu-ro, Jangan-gu, Suwon-si 16419, Gyeonggi-do, Republic of Korea; suhr@skku.edu (J.S.); ey0208@skku.edu (E.O.); 3 Nanoscience and Nanotechnology (International Program/Interdisciplinary), Faculty of Science, Chiang Mai University, Chiang Mai 50200, Thailand; nanthicha581@gmail.com; 4 Office of Research Administration, Chiang Mai University, Chiang Mai 50200, Thailand; thidaratkanthiya05@gmail.com (T.K.); first200294@gmail.com (K.K.); 5 Department of Civil Engineering, Faculty of Engineering, Chiang Mai University, Chiang Mai 50200, Thailand; kannikar@step.cmu.ac.th (K.K.); pitiwat@step.cmu.ac.th (P.W.); 6 Department of Chemistry, Faculty of Science, Chiang Mai University, Chiang Mai 50200, Thailand; patnarin.w@cmu.ac.th; 7 College of Biomedical Engineering, Rangsit University, Pathumthani 12000, Thailand; nuttapol.t@rsu.ac.th; 8 Science and Technology Park (STeP), Chiang Mai University, Chiang Mai 50100, Thailand; 9 Department of Industrial Engineering, Faculty of Engineering, Chiang Mai University, Chiang Mai 50200, Thailand

**Keywords:** fiber, plasma, epoxy, reaction

## Abstract

Antistatic and anti-flame biodegradable polymer composites were developed by melt-blending polybutylene succinate (PBS) with epoxy resin, polybutylene adipate-co-terephthalate (PBAT), and MgO particles. The composite films were prepared using a two-roll mill and an extrusion-blown film machine. Plasma and sparking techniques were used to improve the antistatic properties of the composites. The PBS/E1/PBAT/MgO 15% composite exhibited an improvement in V-1 rating of flame retardancy, indicating an enhancement in the flame retardancy of biodegradable composite films. The tensile strength of the PBS/PBAT blend increased from 19 MPa to 25 MPa with the addition of 1% epoxy due to the epoxy reaction increasing compatibility between PBS and PBAT. The PBS/E1/PBAT and PBS/E1/PBAT blends with MgO 0, 0.5, and 1% showed increases in the contact angle to 80.9°, 83.0°, and 85.7°, respectively, because the epoxy improved the reaction between PBS and PBAT via the MgO catalyst effect. Fourier-transform infrared spectroscopy confirmed the reaction between the epoxy groups of the epoxy resin and the carboxyl end groups of PBS and PBAT by new peaks at 1246 and 1249 cm^−1^. Plasma technology (sputtering) presents better antistatic properties than the sparking process because of the high consistency of the metal nanoparticles on the surface. This composite can be applied for electronic devices as sustainable packaging.

## 1. Introduction

Plastics have become an integral part of modern life owing to their desirable properties, including durability, light weight, corrosion resistance, chemical resistance, low cost, and ease of manufacture [1,2,3]. Plastics are utilized in a wide range of applications, including films, sheets, fibers, yarns, adhesives, particles, and coatings, across various industries such as automotive, biomedical, pharmaceutical, construction, agriculture, electronics, and packaging [4,5]. However, the irreversible ecological damage caused by plastic waste is increasing. This increase in plastic waste has caused environmental harm to both land and marine life. One approach to address this issue is to develop biodegradable polymer blends (BPBs). There are three ways to classify environment-friendly materials: by the origin of the polymer matrix (e.g., natural and synthetic polymers), degradability (e.g., fully, partially, or non-biodegradable), and the content of renewable components (e.g., petroleum-based, partially, or fully bio-based components). Petroleum-derived plastics have led to enormous volumes of environmental pollution owing to their improper disposal. Only 173 million tons of plastic waste are collected for recycling and landfills [6], highlighting the urgent need for new, cost-effective, and degradable plastics that are currently a subject of interest for many academics and industries worldwide.

Poly(butylene succinate) (PBS) is a biodegradable polymer that can be generated from renewable resource-based succinic acid, obtained through the bacterial fermentation of sugars such as glucose, starch, and xylose [7]. Furthermore, the melting point of PBS is considerably lower than those of other commercially available biodegradable polymers, which can reduce the industrial processing time for blending with other materials [8]. The advantages of PBS include its semi-crystalline structure, thermal stability, and ease of forming processes (such as conventional film-casting and blowing techniques), making it an appropriate representative for producing biodegradable films [9]. However, the disadvantages of PBS are its poor ductility and high cost compared with other conventional plastics [10]. Therefore, there is still room for further studies on PBS blends with other polymers. Poly(butylene adipate-co-terephthalate) (PBAT) is a synthetic polymer based on fossil resources that is ductile at room temperature and has good processability [11,12]. PBAT can be blended with PBS to achieve desirable physical, mechanical, and barrier properties. In addition, a high potential for increasing material biodegradability can be achieved by blending PBAT and PBS [13]. Non-thermal plasma is one of the technologies to apply for blending technique [14]. The expectation of blending the high ductility of PBAT with the tensile strength of PBS is to balance the properties of biodegradable film production. However, the blending of polymers has issues regarding their compatibility. The incorporation of a compatibilizer into a polymer blend is pivotal for enhancing the compatibility of the biopolymer blend. Epoxy (E) is a crosslinker that improves the compatibility of polymer blends [15,16]. An epoxy is composed of epoxide groups, which are reactive groups used to crosslink with other functional groups such as hydroxyl and carboxylic groups [17]. This process leads to improved morphology, melting, and crystallization of the polymer blends [18]. Epoxies have the potential to enhance the compatibility of polymer blends.

Electrical and automotive industries require some materials to protect the products before using, such as packaging and coating. Plastic is a candidate material for these applications, while biopolymer has environmental impacts. The limitation of using biopolymers in packaging, electronics, and automotive applications is their potential fire risk and flammability [19]. The addition of various types of flame retardants is a promising and effective way to improve the anti-flaming performance of a blending polymer matrix [20,21,22]. An alternative flame retardant, magnesium oxide (MgO), has been widely used in building materials, fireproof coatings, and refractory materials [23]. MgO is used as an additive for antistatic property [24]. Although MgO can be used as a binder in ceramics and ceramic-matrix composites [25], its use in polymers has not yet been thoroughly investigated.

Atmospheric-pressure plasma is an alternative technique for surface modification that affects surface properties such as chemistry and morphology. Atmospheric-pressure plasma has recently been demonstrated to be an effective technique for modifying polymer surfaces [26]. Only surface properties are changed, but the bulk properties remain unchanged when applying plasma treatment [27]. Dhanumalayan et al. (2017) reported that the average surface roughness increased after plasma treatment [28]. As static charges can destroy sensitive electronic equipment, researchers must develop antistatic materials [29]. Polymeric matrices are the best candidates for antistatic packaging because of their inherent lightness and good processability [30]. Metal particles, such as copper, silver, iron, and carbon allotropes (carbon black, graphite, or carbon nanomaterials), are commonly used as antistatic coatings [31]. The sparking process is an alternative method for generating metal nanoparticles and altering the surface of materials. The advantages of the sparking method include its simplicity, low cost, rapidity, non-vacuum system, and use of non-toxic starting materials (i.e., metal wires) [32,33,34]. The sparking process has the potential to be used as an antistatic additive to improve surface materials. Strengthening the electrostatic resistance of bioplastics using various surface treatment processes, including plasma technology and sparking processes, are two key strategies employed to improve antistatic biodegradable polymer composites. However, there are no previous reports on their use as flame-retardant additives with bioplastics and a comparison of their antistatic abilities with plasma technology and sparking processes. The integrated strategy simultaneously enhances flame retardancy and antistatic performance in biodegradable polymer composites, which expands their potential for being used in advanced sustainable packaging, electronic devices, and automotive components.

Therefore, this study aimed to develop antistatic and anti-flaming biodegradable polymer composites from PBS blends with epoxy resin, PBAT, and MgO to improve their mechanical properties, water resistance, morphology, thermal stability, chemical structure, and flame retardancy. The antistatic properties were improved by plasma technology and a sparking process for coating the film surface. Antistatic and anti-flaming biodegradable polymer composites can be used in packaging, electronics, and automotive applications. MgO was found to improve the reaction and flame retardant of PBS/PBAT blend, while plasma sputtering was an effective technique to improve antistatic property.

## 2. Materials and Methods

### 2.1. Materials

Polybutylene succinate (PBS) pellets (BioPBS™ FD92PM, density 1.24 g/cm^3^, MFI 4 g/10 min at 190 °C, melting point 84 °C, molecular weight of 516.54 g/mol) were purchased from PTT Global Chemical Public Co., Ltd. (Bangkok, Thailand). Polybutylene adipate terephthalate (PBAT) pellets (ECOFLEX F Blend C1200 grade, MFI 2.7–4.9 g/10 min at 190 °C, density 1.26 g/cm^3^, molecular weight of 52.1 kg/mol) were purchased from Unic Technology Co., Ltd. (Chonburi, Thailand). Epoxy resin (E) (diglycidyl ether of bisphenol part A, EPO-TEK 302 grade, molecular weight of 340 g/mol, viscosity 5000–10,000 cPs at 23 °C, glass transition temperature ≥ 40 °C, shore D hardness 73) was purchased from Easy Resin Co., Ltd. (Nonthaburi, Thailand). MgO powder with particle sizes of 0.1–0.5 µm was purchased from Cernic International Co., Ltd. (Nakhon Pathom, Thailand). Titanium, copper, and aluminum wires (0.5 mm diameter) were purchased from Advent Research Materials Co., Ltd. (Oxford, UK). Copper, zinc, aluminum, and titanium oxide (TiO_2_) nanoparticles were purchased from Guangguang Jing Yan Material Co., Ltd. (Xinfeng, China).

### 2.2. Preparation of Biodegradable Polymer Composite

Biodegradable polymer composites were prepared using a melt-blending process due to high shear mixing to disperse metal particles in polymer matrix. Samples were prepared by melt-blending PBS (80%) mixed with 1% epoxy (without hardener), 19% PBAT, and MgO particles at concentrations of 0, 0.5, 1, 2, 5, 10, and 15g, using a two-roll mill (Squeeze 6520 Precision resource Co., Ltd., Bangkok, Thailand) at 130 °C for 10 min. Table 1 lists the composition and nomenclature of the PBS/E1/PBAT/MgO blends. The samples were placed in a mold and compressed into sheets for property assessment using a hot compress at 130 °C for 10 min. All samples were prepared in sheets to evaluate their mechanical properties, thermogravimetric analysis (TGA), differential scanning calorimetry (DSC), water resistance properties, morphological properties, and chemical structure. The best formulation was selected to prepare uniform compound by twin screw extruder, and then prepared to be film by blown film extruder.

### 2.3. Preparation of Biodegradable Polymer Composite Blown Film

The anti-flaming PBS/E1/PBAT/MgO15 composites were mixed using a twin-screw extruder (CTE-D22L32 model, Chareon Tut Co., Ltd., Samutprakarn, Thailand). Before mixing, all materials were dried at 60 °C for 24 h to evaporate moisture content. The extruder temperatures for samples from the hopper to the die were set in sequence as follows, 100 °C, 110 °C, 140 °C, 140 °C, 150 °C, 130 °C, 130 °C, and 120 °C, corresponding to torques of 50% and 70%, with a screw speed of 80 rpm. After mixing, films were produced using an extrusion-blown film machine (Laboratory Film Blowing Machine, Precision resource Co., Ltd., Nonthaburi, Thailand) for antistatic study. The temperature profile was set at 115 °C, 150 °C, 145 °C, 115 °C, and 110 °C from hopper to die, and the screw speed was set at 300 rpm. The film thickness ranged from 0.1 to 0.2 mm. Nanocoated films were produced from these film samples using plasma technology and a sparking process. The antistatic properties were then compared.

### 2.4. Preparation of Plasma Technology (Sputtering) on Biodegradable Polymer Composite Films for Antistatic Properties

The PBS/E1/PBAT/MgO15 composite films were individually functionalized using copper, zinc, aluminum, and titanium oxide (TiO_2_) nanoparticles through DC magnetron sputtering using an MSLV-20020330 system. A high-purity (99.99%) target was secured to the cathode end, placed 5 mm from the specimen’s surface. The vessel was evacuated prior to sputtering and filled with gas. The argon gas pressure was maintained at 1.0 × 10^−1^ Torr/m^3^ in the chamber, and the argon gas flow was stabilized at 5 sccm. The oxygen (O_2_) gas pressure was set at 3.0 × 10^−1^ Torr/m^3^. The substrate underwent smooth deposition of metal nanoparticles using DC magnetron sputtering powered by a 100 W DC power supply (RPG-50 reactive plasma generator) for the sputtering process. The coating duration was 3 min, and the coating process was applied to only one side of the PBS/E1/PBAT/MgO15 composite film. After sputtering, the final PBS/E1/PBAT/MgO15 composite film was extracted from the chamber to measure its physical, chemical, and electrical characteristics.

### 2.5. Preparation of Nano-Metal-Particles (NMP) Sparking Process on Biodegradable Polymer Composite Films for Antistatic Properties

Ti, Cu, and Al wires (0.5 mm diameter) were purchased from Advent Research Materials Co., Ltd. (Oxford, UK). The two sharp tips (20 mm long) of the metal wire were connected to the anode and cathode of the sparking machine. The tips were aligned 2 mm above the PBS/E1/PBAT/MgO composite film (5 cm × 10 cm × 0.1 cm) with a 1 mm gap between the anode and cathode. The PBS/E1/PBAT/MgO composite film was individually sparked with Ti, Cu, and Al at 3 kV and 3 mA, and the tip holders of the wires were moved along the XY axis at a speed of 100 mm/min. The number of repetitions for each metal wire is listed in Table 2.

### 2.6. Flame-Retardant Characterization

The flame retardancy of the samples was tested using the UL94 standard [35] (size: W12.7 mm × L127 mm × T1 mm) for vertical burns (V-0 to V-2 Rating). A burner flame was applied to the free end of the specimen for two 10 s intervals, separated by the time it took for the flaming combustion to cease after the first application. Five specimens were tested. The classifications of the UL94 standards [35] are presented in Table 3.

### 2.7. Mechanical Properties

The mechanical and tensile properties of the anti-flaming biodegradable polymer composites were evaluated. The tensile properties were measured using a tensile tester (Model MCT–1150, Tokyo, Japan) at a distance of 10 mm and a crosshead speed of 20 mm/min. The specimens, prepared as bone-shaped samples according to the JIS K 6251-7 standard [36], were tested with a minimum of five specimens for each composition. The specimens were conditioned at 25 °C with 50 ± 2% RH for 24 h. The maximum force and elongation at breaking were recorded.

### 2.8. Thermogravimetric Analysis (TGA)

Thermogravimetric analysis of all samples was performed using a thermogravimetric analyzer (Mettler Toledo STARe system TGA/DSC3+, Greifensee, Switzerland). Each sample, weighing approximately 5–10 mg, was heated from 25 °C to 600 °C at a heating rate of 15 °C/min under N_2_.

### 2.9. Differential Scanning Calorimetry (DSC)

Samples weighing 5–10 mg were placed in an alumina pan. The temperature program involved heating from −50 °C to 200 °C, cooling from 200 °C to −50 °C, and further heating from −50 °C to 200 °C, all at a constant heating rate of 10 °C/min under a nitrogen atmosphere. The crystallinities (%X_c_) of the samples were calculated using Equation (1).

(1) $$\mathrm{Crystallinity}\;\%\;(\%Xc)=(\frac{{∆H}_{m}-{∆H}_{c}}{\omega H_{0}^{m}})\;\times \;100$$

where ∆H_m_ and ∆H_c_ are the enthalpies of melting and cold crystallization, respectively, and ω and ∆H^0^_m_ are the weight fraction of PBS and PBAT and the melting enthalpy of 100% pure polymer (110 and 114 J/g), respectively.

### 2.10. Morphological Properties

The cross-sectional morphologies of the films were examined using an SEM (FE-SEM, JSM-IT800 series, JEOL Co., Ltd., Tokyo, Japan). The samples, measuring 5 mm × 30 mm × 1 mm (width × length × thickness), were placed in liquid nitrogen for 2 min and then fractured to obtain the cross-sectional surfaces. All the fractured sample surfaces were vacuum-coated with a thin gold layer and observed at 15 kV. The morphology was characterized based on the fracture surfaces of the samples.

### 2.11. Water Contact Angle

Water contact angle measurements were performed using a drop shape analyzer (DSA30E, Krüss Co., Ltd., Hamburg, Germany). The samples were prepared as sheets measuring 20 × 20 × 1 mm (width × length × thickness) and fixed on a glass slide. Water was dropped onto the surface of the sheets, and images were automatically recorded every minute for 10 min. The water contact angle was then measured using image analysis after droplet formation.

### 2.12. Chemical Structure Characterization by Fourier-Transform Infrared (FTIR) Spectroscopy

The chemical structure was characterized using FTIR spectroscopy (FT/IR–4700, Jasco Corp., Tokyo, Japan). The samples were prepared as films. FTIR spectra were recorded between 4000 and 400 cm^−1^ with a resolution of 4 cm^−1^ and 64 scans. The chemical structure was identified by IR spectroscopy.

### 2.13. Determine the Antistatic Properties of the Plasma Technology and Nano-Metal-Particles (NMP) Sparking Method on Biodegradable Polymer Composite Films

The surface resistance of the biodegradable polymer composite films, improved by two types of surface treatments (plasma technology and NMP sparking method), was determined using an electrostatic field meter (ARS-H002ZA series, Korea DONG IL TECHNOLOGY Co., Ltd., Hwaseong-si, Gyeonggi-do, Korea). The surface static electricity of the films was tested under conditions of 24 ± 2 °C and 40 ± 2% RH, at a distance of 25 mm.

### 2.14. Statistical Analysis

The results were analyzed using one-way analysis of variance (ANOVA) with SPSS software (version 17, Armonk, NY, USA). Differences in tensile strength and water contact angle were estimated using Duncan’s test, with significance set at *p* < 0.05.

## 3. Results and Discussion

### 3.1. Chemical Structure

**Figure 1:**
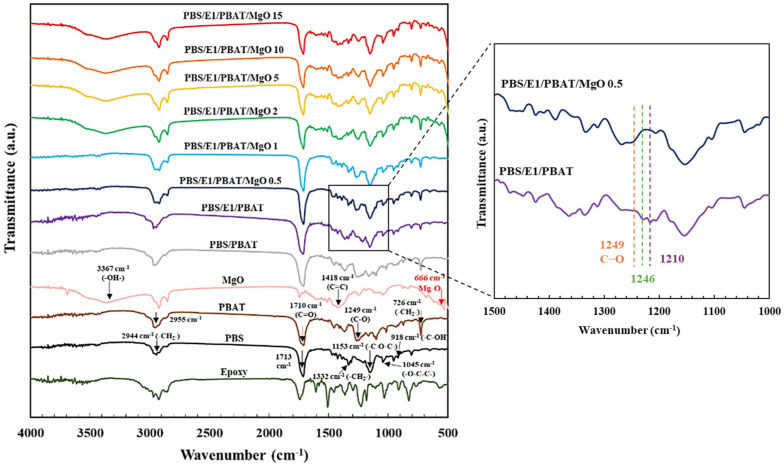
FTIR spectra of PBS, PBAT, PBS/PBAT, PBS/E1/PBAT, and PBS/E1/PBAT/MgO 0.5–15%.

Figure 1 presents the FTIR spectra of PBS, PBAT, PBS/PBAT, PBS/E1/PBAT, and PBS/E1/PBAT/MgO at concentrations ranging from 0.5 to 15%. The chemical structures of the blends were analyzed using FTIR spectroscopy. The epoxy exhibited characteristic peaks at 914 and 1670 cm^−1^, corresponding to the absorption of epoxy groups and the C=C stretching bands of aromatic rings, respectively [37]. The peaks at 1455, 1508, and 1606 cm^−1^ are attributed to the CH_3_ asymmetrical bending and the C–C stretching vibrations of the aromatic rings [38]. The PBS peak at 918 cm^−1^ corresponds to –C–OH bending in the carboxylic acid groups. The band at 1045 cm^−1^ was attributed to the –O–C–C– stretching vibrations in PBS. Peaks in the range of 1153 cm^−1^ were linked to the stretching of the –C–O–C bonds in the ester linkages of PBS. The band at 1713 cm^−1^ was associated with the C=O stretching vibrations of the ester groups, while the peaks at 1332 and 2944 cm^−1^ were assigned to the symmetric and asymmetric deformational vibrations of the –CH_2_– groups in the main PBS chains [39,40,41]. PBAT exhibits a peak at 726 cm^−1^, associated with the vibrations of the –CH_2_– groups adjacent to the methylene groups of the polymer backbone. The peaks at 2955 cm^−1^ are attributed to the symmetric stretching vibrations of the –CH– groups, while the peaks at 1249 and 1710 cm^−1^ were linked to the stretching of the C–O bonds from aliphatic groups and carbonyl groups (C=O) in the ester linkage, respectively [42,43]. MgO displayed peaks at 409 and 666 cm^−1^, indicating the vibration of Mg–O in the MgO particles. The band at 1418 cm^−1^ was due to C=C stretching vibrations, while the peak at 3367 cm^−1^ was assigned to the OH groups in the MgO particles [44,45]. The PBS/PBAT with 1% epoxy exhibited combined spectra of PBS, PBAT, and epoxy resin. A peak intensity at 1249 cm^−1^ (C–O stretching groups) decreased, indicating a change in the C–O vibration due to the reaction between the carboxyl end groups of PBS and PBAT with epoxy. A decrease in the peak intensity of the C–O vibration due to the epoxy resin has been previously reported [46]. The PBS/E1/PBAT with 0.5–15% MgO exhibited combined characteristics of PBS, PBAT, epoxy, and MgO spectra. In PBS/E1/PBAT/MgO0.5, two new peaks at 1246 and 1249 cm^−1^ were observed, with a decrease in the peak at 1210 cm^−1^. The intensity of the peak at 1249 cm^−1^ decreased, followed by a reduction in the 1246 cm^−1^ peak intensity with increasing MgO content. These two new peaks (1246 and 1249 cm^−1^) and the reduction in peak intensity (1210, 1246, and 1249 cm^−1^) indicated the reaction between the COOH groups of PBS and PBAT with the epoxy groups of epoxy, accelerated by the MgO catalyst. Crosslinking within the PBS and PBAT phases and interfacial crosslinking of PBS/PBAT was observed, which improved its properties.

### 3.2. Morphology

**Figure 2:**
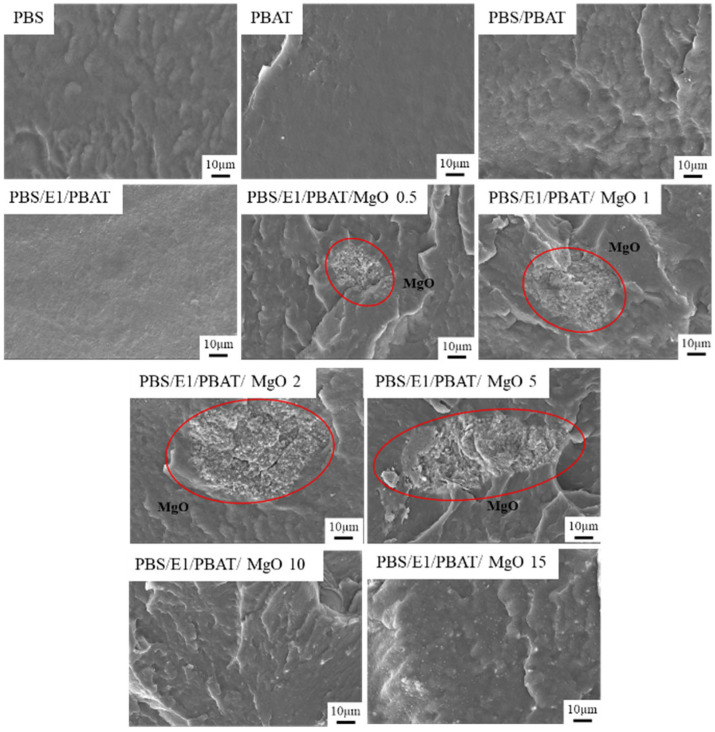
Scanning electron micrographs of PBS, PBAT, PBS/PBAT, PBS/E1/PBAT, and PBS/E1/PBAT/MgO 0.5–15%.

**Figure 3:**
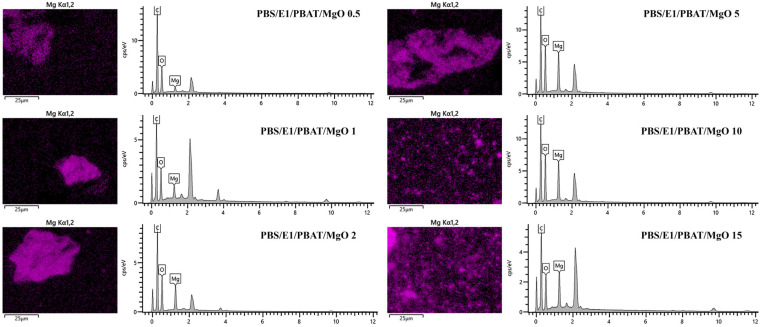
EDS mode of PBS/E1/PBAT/MgO 0.5–15%.

Figure 2 displays the SEM images of the fracture surfaces of PBS, PBAT, PBS/PBAT, PBS/E1/PBAT, and PBS/E1/PBAT with 0.25–5% MgO blends. PBAT exhibited a smooth fracture surface, while PBS showed a slightly rough fracture surface. The PBS/PBAT blend exhibited increased roughness due to phase separation incompatibility between PBS and PBAT. This phase separation results in non-uniform cross-sections in polymer blends [13]. Meanwhile, PBS/E1/PBAT demonstrated an increase in the smoothness of the fracture surface and improved dispersion of epoxy around the materials because the epoxy enhanced the compatibility of the PBS/PBAT blend. This compatibility was enhanced owing to increased interfacial adhesion between the two phases [47]. Adding MgO at concentrations of 0.5–5% in PBS/E1/PBAT blends resulted in increased roughness of the fracture surfaces with the MgO content, due to the aggregation of MgO particles in the samples. Epoxy of samples was indicated to form interactions with MgO, which induced agglomeration of MgO. These aggregates can affect to mechanical properties as a big defect for polymer matrix. Aggregation of metal nanoparticles has been previously reported [48]. However, adding MgO at concentrations of 10 and 15% resulted in smoother surfaces due to the well-dispersed MgO and smaller-sized particles, which improved the elongation at break of the PBS/E1/PBAT/MgO 10 and 15% blends. As previously reported, the mechanical properties of a polymer composite are enhanced by the dispersion and size of the particles in the matrix [49]. Figure 2 also includes SEM-EDS images showing the dispersion of MgO on the fracture surfaces of PBS/E1/PBAT with 0.5–15% MgO blends. In these SEM-EDS images (Figure 3), the PBS/E1/PBAT with 0.5–5% MgO blends showed agglomeration of large MgO particles within the matrix, which adversely affected the distribution of MgO particles and decreased the mechanical properties of the blends. The addition of 10–15 wt.% MgO demonstrated increased dispersion within the matrix, indicating an improvement in the elongation at break of the blends. Big aggregates were indicated during the first state of mixing, while aggregates crushing by high shear force induced fine MgO particle distribution in PBS/E1/PBAT/MgO 10 and 15% blends. Furthermore, the interaction between the carboxylic groups and Mg^2+^ ions increased the elongation at break. As previously reported, enhanced dispersion of MgO and interaction of metal ions contribute to an increase in elongation at break [50,51].

### 3.3. Mechanical Properties

**Figure 4:**
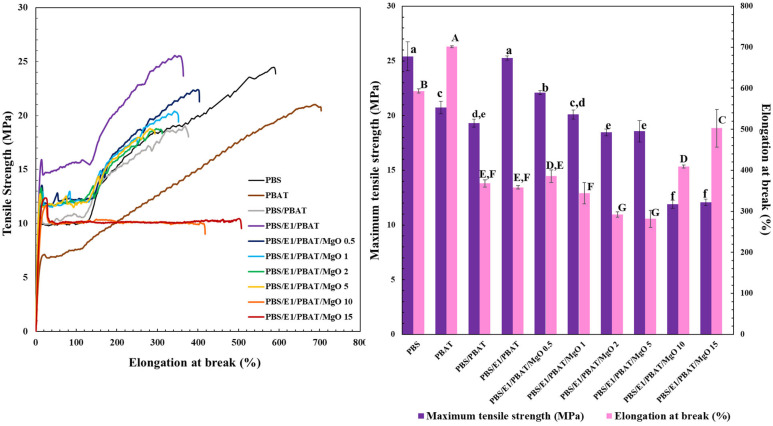
Tensile properties of PBS, PBAT, PBS/PBAT, PBS/E1/PBAT, and PBS/E1/PBAT blends with 0.5–15% MgO. Mean values of elongation at break (lowercase letters) and maximum tensile strength (uppercase letters) differ significantly (*p* < 0.05).

The tensile properties of PBS, PBAT, PBS/PBAT, PBS/E1/PBAT, and PBS/E1/PBAT/MgO at concentrations ranging from 0.5 to 15% are presented in Figure 4. The tensile strength of PBS (25 MPa) was higher than that of PBAT (20 MPa). However, the elongation at break of PBS (593%) was lower than that of PBAT (701%). The PBS/PBAT blend showed tensile strength and elongation at break of 19 MPa and 367%, respectively. The mechanical properties of the PBS/PBAT blend decreased due to the phase separation between PBS and PBAT, indicating the incompatibility of the polymer blend. Phase separation in polymer blends leading to reduced tensile strength has been previously reported [52,53]. Upon adding 1% epoxy to PBS/PBAT, the tensile strength increased to 25 MPa, and the elongation at break decreased to 358%, due to increased compatibility between PBS and PBAT facilitated by the epoxy reaction. The functional groups of the polymer blends reacted between the carboxyl end groups of PBS and PBAT and the epoxy groups. Improvement in the tensile strength of polymer blends due to the reaction with epoxy groups has been previously documented [54,55]. The addition of 0.5 to 15% MgO to PBS/E1/PBAT resulted in reduced tensile strength compared to the PBS/E1/PBAT blend. PBS/E1/PBAT/MgO 0.5 exhibited the highest tensile strength (22 MPa), which was similar to low density polyethylene (10–18 MPa) [56,57,58]. The addition of 0.5–5% MgO to PBS/E1/PBAT decreased the tensile strength and elongation at break due to the presence of agglomerates and large particles, which reduced compatibility and acted as defects in the PBS/E1/PBAT/MgO blends. The agglomerated particles affected the interfacial adhesion and reduced the tensile strength [59]. The addition of 5–15% MgO showed the decreasing tensile strength due to the excessive amount of nanoparticles with low interfacial interaction between MgO and polymers surface, which induced MgO particle agglomeration as a big defect to limit the polymer stress distribution [60]. However, the elongation at break of PBS/E1/PBAT/MgO 10–15% increased to 415.9% and 505.2%, respectively, because of better dispersion of MgO. Furthermore, the high dispersion of MgO can be attributed to the high shear forces during blending, which promotes effective particle dispersion. The interaction of the carboxylic and hydroxyl groups with Mg^2+^ ions and/or epoxy groups causes plasticization properties. An increase in elongation at break due to the uniform dispersion of MgO and the interaction of metal ions in the polymer matrix has been previously reported [50,51].

### 3.4. Differential Scanning Calorimetry (DSC)

**Figure 5:**
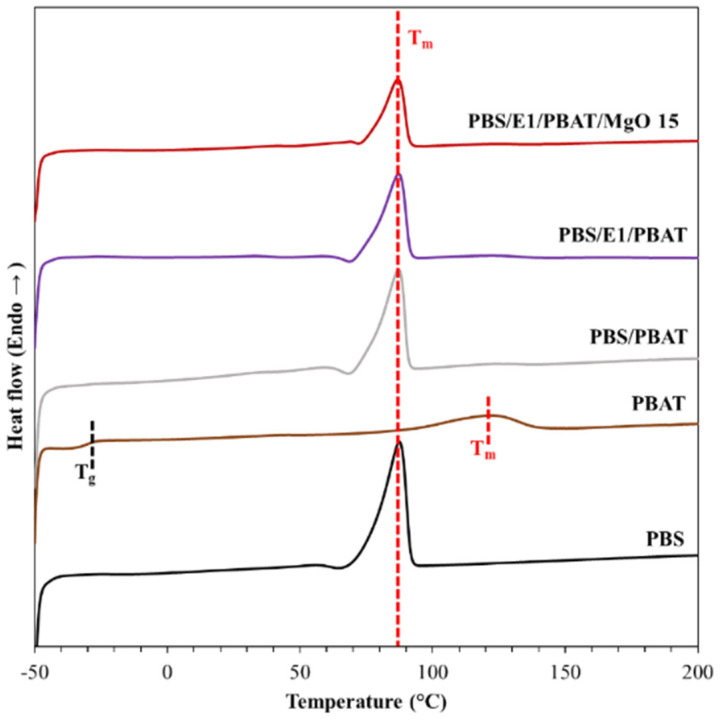
DSC curves of the second scan for PBS, PBAT, PBS/PBAT, PBS/E1/PBAT, and PBS/E1/PBAT/MgO15.

DSC curves were utilized to determine the thermal properties of the PBS/E1/PBAT/MgO blends. Figure 5 displays the second scans of the DSC curves for PBS, PBAT, PBS/PBAT, PBS/E1/PBAT, and PBS/E1/PBAT/MgO15. PBAT exhibited a glass transition temperature (T_g_) of −28 °C and a melting temperature (T_m_) of 121 °C. The T_m_ of PBS was recorded at 87 °C, while its T_g_ was not observed during this measurement (Table 4). The glass transition and melting temperatures of PBS and PBAT have been previously reported [9,61]. The T_m_ of PBS/PBAT, PBS/E1/PBAT, and PBS/E1/PBAT/MgO15 were recorded at 87, 87, and 86 °C, respectively. The blends exhibited a decrease in T_m_ compared to pure PBS. The crystallinities of PBS, PBAT, PBS/PBAT, PBS/E1/PBAT, and PBS/E1/PBAT/MgO15 were 43%, 11%, 26%, 29%, and 19%, respectively. Crystallinities of PBS/PBAT, PBS/E1/PBAT, and PBS/E1/PBAT/MgO15 were PBS crystal which were calculated from the melting peak of PBS. However, recrystallization was not observed in any of the blends due to the high content of existing crystals without nucleation sites to induce new crystallization.

### 3.5. Thermogravimetric Analysis (TGA)

**Figure 6:**
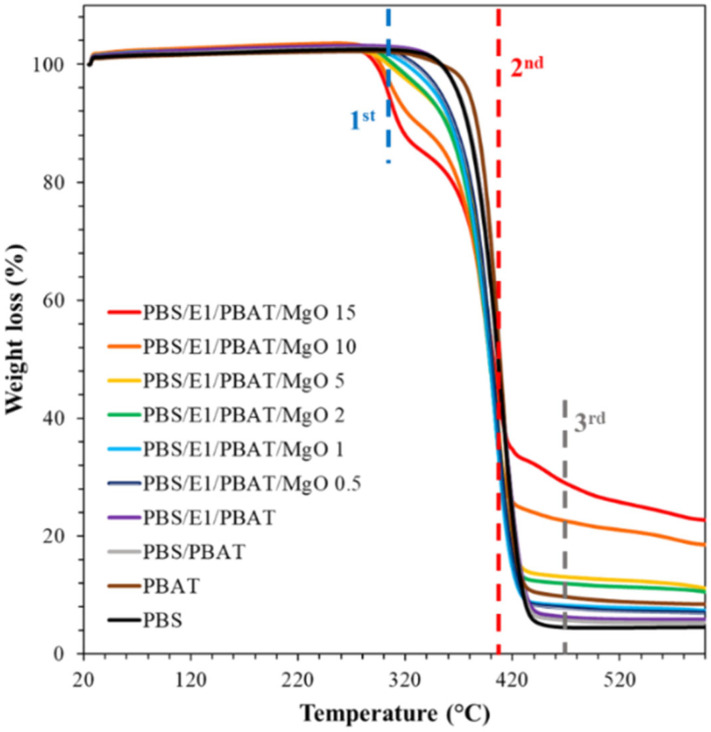
TGA thermograms of PBS, PBAT, PBS/PBAT, PBS/E1/PBAT, and PBS/E1/PBAT/MgO ranging from 0.5 to 15%.

Figure 6 presents the TGA thermograms of PBS, PBAT, PBS/PBAT, PBS/E1/PBAT, and PBS/E1/PBAT/MgO ranging from 0.5 to 15%. PBS, PBAT, PBS/PBAT, and PBS/E1/PBAT exhibited one main weight-loss stage and a corresponding heat-flow peak. The primary thermal degradation temperatures for PBS and PBAT were approximately 400 and 413 °C, respectively. This thermal degradation has been documented in previous studies [62,63]. The PBS/PBAT blend showed a weight-loss stage at around 400 °C, similar to pure PBS. However, the weight-loss stage for PBS/E1/PBAT slightly increased to 410 °C, indicating enhanced thermal stability due to the crosslinking of PBS, PBAT, and epoxy resin. The degradation temperature of epoxy resin ranged between 400 and 600 °C, which contributes to the increased degradation temperature of the blends by cross-linking [54,64]. In the TGA thermogram of PBS/E1/PBAT/MgO 0.5–15%, the main step in weight loss occurred in the range of 398–400 °C, which is associated with the depolymerization of the polymer catalyzed by MgO. However, the addition of MgO at 2–15% introduced multiple-stage weight loss, observed at two stages for PBS/E1/PBAT/MgO 2 and 5%. The first stage, occurring between 310 and 320 °C, indicates the dehydroxylation of the surface of MgO particles within the polymer composites. The dehydroxylation of MgO surfaces has been previously been reported [44]. For PBS/E1/PBAT/MgO 10–15%, there were three stages: the first at 310–320 °C, the second at 390–400 °C, and the third at 450–460 °C, the latter being a heat transfer stage attributed to the thermal degradation of the MgO particles. The degradation temperature of the MgO particles has been reported in earlier studies [65]. Additionally, the residue percentages at 600 °C for PBS/E1/PBAT/MgO 10–15% were recorded at 19% and 23%, respectively. The increase in residue percentage could be attributed to the presence of inorganic MgO particles and their high thermal stability.

### 3.6. Water Contact Angles

**Figure 7:**
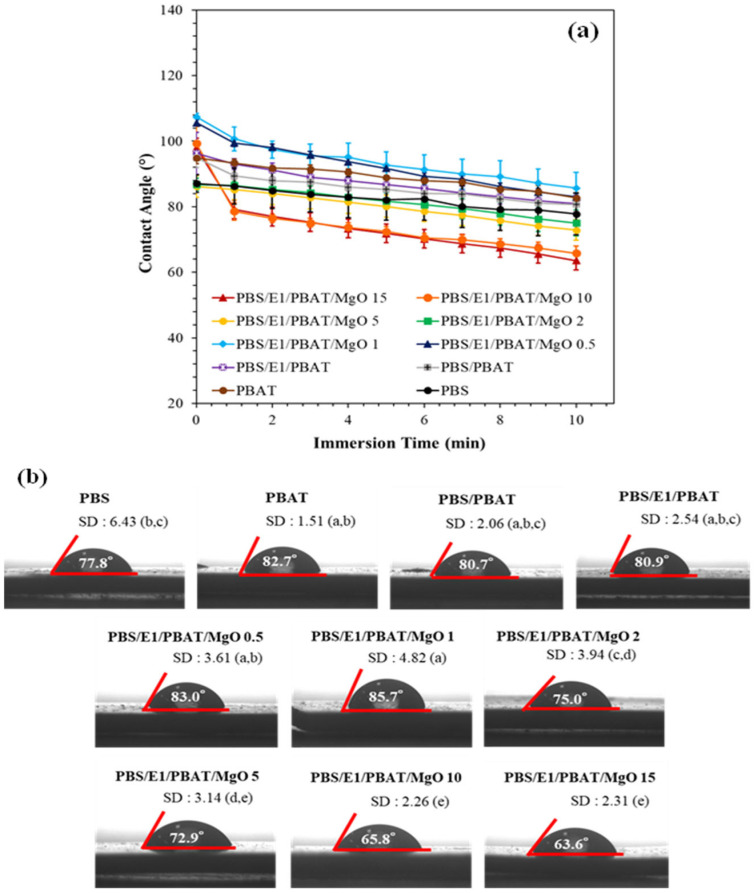
Water contact angles of PBS, PBAT, PBS/PBAT, PBS/E1/PBAT, and PBS/E1/PBAT/MgO 0.5–15% (**a**) plots of samples and (**b**) values at 10 min.

The water contact angle is commonly used to determine the hydrophilic and hydrophobic properties of surfaces. Typically, hydrophobic materials exhibit large water contact angles. Figure 7 shows the water contact angles for PBS, PBAT, PBS/PBAT, PBS/E1/PBAT, and PBS/E1/PBAT/MgO at 0.5–15%. The water droplet was absorbed onto the surface, and the contact angle was automatically recorded every minute for 10 min. PBS exhibited a contact angle of 77.8° after 10 min, whereas PBAT exhibited a higher angle of 82.7°, reflecting its more hydrophobic nature despite PBS generally being more hydrophilic [66]. The contact angle for the PBS/PBAT blend was 80.7°, higher than that of pure PBS, indicating that the hydrophobicity of PBAT enhanced the water resistance of the blends. Adding epoxy 1% to PBS/PBAT increased the contact angle to 80.9°, suggesting improved compatibility of the blend, which has been linked to increasing water contact angles in previous studies [67]. For the PBS/E1/PBAT blends with 0.5% and 1% MgO, the water contact angles at 10 min increased to 83.0° and 85.7°, respectively. This increase indicates that the addition of 1% MgO to the PBS/E1/PBAT blend increased the surface tension and hydrophobicity due to the metal particles and the increased density of the composites [68,69]. However, adding MgO at 2%, 5%, 10%, and 15% decreased the water contact angle at 10 min to 75.0°, 72.9°, 65.8°, and 63.6°, respectively, owing to increase in roughness of the composite films’ surfaces and the hydrophilic nature of alkaline MgO [70]. The increasing MgO content exhibited hygroscopic properties and reacts with water to form Mg(OH)_2_, which reduced hydrophobic nature of composites [71]. The decreasing water contact angle in polymer blend with MgO content has also been reported [72,73,74,75].

### 3.7. Flame-Retardant Characterization

The flammability of the samples was evaluated using the UL94 standard Vertical Burning test. The UL-94 ratings for PBS, PBAT, PBS/PBAT, and PBS/E 0.5–20/PBAT samples are presented in Table 5. PBS, PBAT, PBS/PBAT, and PBS/E1/PBAT all exhibited a V-2 flame retardancy rating, where flaming drips were observed after burning but did not reach the holding clamp. The addition of 0.5% to 2% MgO to PBS/E1/PBAT extended the burning times to over 30 s for T1, and 250 s for T1 + T2. Adding 5% and 15% MgO continuously improved flammability, with T1 times of 12 s and T1 + T2 times of 128, 125, and 122 s. Specifically, PBS/E1/PBAT/MgO 15 achieved a V-1 degree of flame retardancy, indicating that the drips from the samples did not ignite the cotton batting below, which reflects a significant improvement in flame retardancy of the biodegradable composite films due to the addition of MgO, enhancing self-extinguishing properties after the removal of the flame. V-1 rating classifications that indicate enhanced flame retardancy have been documented in previous studies [72,76]. MgO acted as both a catalyst and a flame-retardant agent, helping to form a crosslinked char that was firmly fixed on top of the composite, subsequently restricting and extinguishing the flame [77].

### 3.8. Antistatic Properties of the Plasma Technology (Sputtering) and Nano-Metal-Particles (NMP) Sparking Process Method for Coating on Biodegradable Polymer Composite Films

**Figure 8:**
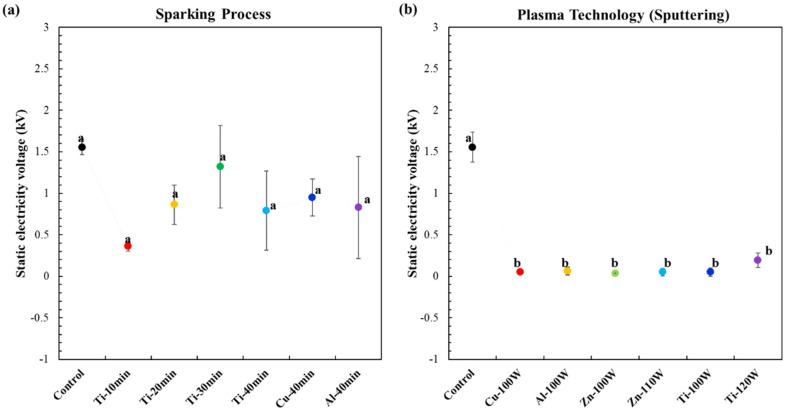
Effects of coating method on antistatic properties of the biodegradable polymer composite films: (**a**) sparking process; (**b**) plasma technology (sputtering).

Figure 8 shows static electricity voltage of the coated biodegradable polymer composite films using sparking process and plasma technology (sputtering). Plasma technology involved DC magnetron sputtering at 100, 110, and 120 W for sputter-coating with Ti, Cu, Zn, and Al nanoparticles. The uncoated (control) PBS/E1/PBAT/MgO15 film exhibited a high voltage of 1.56 kV, indicating poor antistatic properties. The voltages of all coated films decreased with each method applied. For the PBS/E1/PBAT/MgO15 films coated using the sparking process, the surface voltages were measured at 0.364 kV (Ti-10 min), 0.864 kV (Ti-20 min), 1.32 kV (Ti-30 min), 0.79 kV (Ti-40 min), 0.948 kV (Cu-40 min), and 0.83 kV (Al-40 min). Moreover, the surface voltages of PBS/E1/PBAT/MgO15 films coated with plasma technology were significantly reduced to 0.05 kV (Cu-100 W), 0.062 kV (Al-100 W), 0.036 kV (Zn-100 W), 0.05 kV (Zn-110 W), 0.05 kV (Ti-100 W), and 0.192 kV (Ti-120W). The film’s antistatic properties are enhanced due to the conducting properties of the metal particles and oxides coated on it. Conductive additives, typically used with polymers, contribute to antistatic properties [78,79,80,81] by facilitating the movement of ions on the substrate surface [82]. Films coated using plasma technology exhibited lower voltage levels compared to those coated using the sparking process. Plasma sputtering involves accelerating electrons under an electric field until they collide with the atoms of the target material, leading to the breakdown of these atoms into positive ions. These ions are then accelerated towards the cathode, where they collide with the coating material particles, causing deposition onto the substrate positioned near the anode. The plasma process, resulting from the ionization of neutral gases [83,84,85], operates at the ion level, which enhances the nanoparticle dispersion ability on the surface of the film. In contrast, the sparking process produces metal nanoparticles by bombardment with electrons and ions, leading to a less uniform nanoparticle distribution on the surface compared to the plasma process [34]. Therefore, plasma technology is more effective in improving the antistatic properties due to the finer particle distribution and higher uniformity of the metal nanoparticles on the film surface.

## 4. Conclusions

Antistatic and anti-flaming biodegradable polymer composite films were successfully developed through the melt blending of PBS/PBAT and epoxy with MgO. FTIR analysis confirmed the reaction between the –COOH groups of PBS and PBAT and the epoxy groups. The PBS/E1/PBAT/MgO 15 blends exhibited a fine MgO particle distribution within the PBS/E1/PBAT matrix. The tensile strength of PBS/E1/PBAT was increased to 25.3 MPa, while the elongation at break of PBS/E1/PBAT/MgO 15 was raised to 505.2% compared to PBS/PBAT. This improvement in properties was due to the reaction of the epoxy in the PBS/E1/PBAT blends. The addition of MgO to the PBS/E1/PBAT blends enhanced their thermal decomposition behavior and water resistance, attributable to the high thermal stability and hydrophobicity of the metal particles. MgO acted as a catalyst for the –COOH and epoxy to accelerate reactions. The flame-retardant characteristics of the PBS/E1/PBAT/MgO 15 blends improved to a V-1 degree of flame retardancy. MgO served as a flame retardant, enhancing the strength of the residual char from the crosslinked matrix. The antistatic properties of the films were improved using both plasma technology and sparking methods. The antistatic effect of the plasma sputtering process proved superior to that of the sparking process because the sputtering method enhanced the uniform dispersion of metal nanoparticles on the film’s surface. Anti-flaming and antistatic biodegradable polymer composites of PBS/E1/PBAT/MgO with enhanced properties hold potential for use in packaging, electronics, and automotive applications. It can be used as an environmentally friendly insulation or electrical circuit board packaging application. These polymer composites can replace petroleum polymer insulators and electronic devices packaging film.

## Figures and Tables

**Table 1:** Composition of PBS/E1/PBAT/MgO blends.

Samples	Composition (g)	
	PBS/E1/PBAT	MgO
1. PBS/E1/PBAT	100	-
2. PBS/E1/PBAT/MgO 0.5	99.5	0.5
3. PBS/E1/PBAT/MgO 1	99	1
4. PBS/E1/PBAT/MgO 2	98	2
5. PBS/E1/PBAT/MgO 5	95	5
6. PBS/E1/PBAT/MgO 10	90	10
7. PBS/E1/PBAT/MgO 15	85	15

**Table 2:** Repeated time of each metal wire.

No.	Metal Wire: Repeated Times
1	Control (Untreated)
2	Titanium (Ti:10)
3	Titanium (Ti:20)
4	Titanium (Ti:30)
5	Titanium (Ti:40)
6	Copper (Cu:40)
7	Aluminum (Al:40)

**Table 3:** Classification of UL94V standards.

UL 94 Test (Vertical Burning Test)			
Test Criteria	V-0	V-1	V-2
Burning time for each individual test specimen (s) (after first and second flame applications T_1_ or T_2_)	≤10 s	≤30 s	≤30 s
Total burning time (s) (T_1_ + T_2_)	≤50 s	≤250 s	≤250 s
Dripping of burning specimen (ignition of cotton batting)	No	No	Yes
Combustion up to holding clamp (specimens completely burned)	No	No	No

**Table 4:** Second scan DSC results for PBS, PBAT, PBS/PBAT, PBS/E1/PBAT, and PBS/E1/PBAT/MgO 15.

Sample	T_g_ (°C)	T_c_ (°C)	T_m_ (°C)	ΔH_m_ (J/g)	ΔH_c_ (J/g)	ΔX_c_ (%)
PBS	-	-	87.5	47.1	-	42.8
PBAT	−28.2	-	121.5	12.9	-	11.3
PBS/PBAT	-	-	87.1	33.9	-	26.3
PBS/E1/PBAT	-	-	87.1	31.4	-	28.6
PBS/E1/PBAT/MgO15	-	-	86.8	21.0	-	19.1

**Table 5:** Anti-flame ability of PBS, PBAT, PBS/PBAT, PBS/E1/PBAT, and PBS/E1/PBAT blend with MgO at 0.5–15%.

Samples	Class UL 94 (V0–V2)	Ignition (Flaming Drip) (Yes/No)	Specimen Burns up to Holding Clamp. (Yes/No)
PBS	V-2	Yes	No
PBAT	V-2	Yes	No
PBS/PBAT	V-2	Yes	No
PBS/E1/PBAT	V-2	Yes	No
PBS/E1/PBAT/MgO 0.5	-	Yes	No
PBS/E1/PBAT/MgO 1	-	Yes	No
PBS/E1/PBAT/MgO 2	-	Yes	No
PBS/E1/PBAT/MgO 5	V-2	Yes	No
PBS/E1/PBAT/MgO 10	V-2	Yes	No
PBS/E1/PBAT/MgO 15	V-1	No	No

## Data Availability

The datasets used and/or analyzed during the current study are available from the corresponding author on reasonable request.
